# Chronic Sublethal Aluminum Exposure and *Avena fatua* Caryopsis Colonization Influence Gene Expression of *Fusarium avenaceum* F.a.1

**DOI:** 10.3389/fmicb.2020.00051

**Published:** 2020-02-04

**Authors:** Ricky W. Lewis, Patricia A. Okubara, E. Patrick Fuerst, Ruifeng He, David Gang, Tarah S. Sullivan

**Affiliations:** ^1^Department of Crop and Soil Sciences, Washington State University, Pullman, WA, United States; ^2^Wheat Health, Genetics, and Quality, USDA-ARS, Pullman, WA, United States; ^3^Institute of Biological Chemistry, Washington State University, Pullman, WA, United States

**Keywords:** wild oat, *Fusarium*, weed seed decay, fungal siderophore, soil acidification, sublethal aluminum toxicity, plant pathogens, soil microbiology

## Abstract

*Fusarium avenaceum* F.a.1 is a novel strain of a fungal plant pathogen capable of preferentially decaying wild oat (*Avena fatua*) caryopses compared with those of wheat (*Triticum aestivum*). Understanding the molecular mechanisms governing weed seed-pathogen interactions is crucial to developing novel weed seed suppression technologies. Additionally, wild oat often competes with wheat in regions undergoing soil acidification, which leads to increases in soluble concentrations of many metals, including aluminum (Al). There is a dearth of information regarding the gene expression responses of *Fusarium* species to Al toxicity, or how metal toxicity might influence caryopsis colonization. To address this, a transcriptomic approach was used to investigate molecular responses of F.a.1 during wild oat caryopsis colonization in the presence and absence of chronic, sublethal concentrations of Al (400 μM). Caryopsis colonization was associated with induction of genes related to virulence, development, iron metabolism, oxidoreduction, stress, and detoxification, along with repression of genes associated with development, transport, cell-wall turnover, and virulence. Caryopsis colonization during Al exposure resulted in the induction of genes associated with virulence, detoxification, stress, iron metabolism, oxidoreduction, and cell wall turnover, along with repression of genes associated with cell wall metabolism, virulence, development, detoxification, stress, and transcriptional regulation. Aluminum exposure in the absence of caryopses was associated with induction of genes involved in siderophore biosynthesis, secretion, uptake, and utilization, along with several other iron metabolism-related and organic acid metabolism-related genes. The siderophore-related responses associated with Al toxicity occurred concurrently with differential regulation of genes indicating disruption of iron homeostasis. These findings suggest Al toxicity is attenuated by siderophore metabolism in F.a.1. In summary, both caryopsis colonization and Al toxicity uniquely influence transcriptomic responses of F.a.1.

## Introduction

Agronomic weeds are a global issue that result in billions of dollars in annual economic losses (Pimentel et al., 2001). Weed seeds exist in high densities in soils, and may persist for many years due to long-term dormancy and decay resistance [as reviewed by Pollard (2018)]. Promoting microbial-driven seed decay is a potential ecological approach to long-term weed management by depleting the weed seedbank. In temperate regions of the world, including wheat-growing regions, wild oat (*Avena fatua*) is considered one of the ten worst weeds (Beckie et al., 2012). A major factor contributing to the persistence of *A. fatua* is that seeds can remain dormant in the soil for many years, thus generating a large soil seedbank that can readily develop herbicide resistance (Beckie et al., 2012).

Work by de Luna et al. (2011) resulted in hundreds of soil fungi isolates from dormant wild oat seeds, and it was found that *Fusarium avenaceum* isolate F.a.1 elicited the most rapid and pronounced decay of wild oat seeds. Successive studies *in vitro* showed F.a.1 is capable of preferentially decaying *A. fatua* compared with wheat caryopses (seeds without hulls), and that the fungus induces activity of several defense enzymes, including polyphenol oxidase, chitinase, and peroxidase, in both whole caryopses and caryopsis leachates (the soluble enzyme fraction) (Anderson et al., 2010; Fuerst et al., 2011, 2014, 2018). The latest work showed that incubation of wheat and wild oat seeds on a F.a.1 fungal mat resulted in a rapid increase in decay rating of wild oat, while wheat seeds germinated (Fuerst et al., 2018). F.a.1 exposure also resulted in increased polyphenol oxidase in wild oat and wheat caryopses, though the increase was 3.4 times that of the pathogen-free control in wild oat, and 1.8 times in wheat (Fuerst et al., 2018).

In addition to weed pressure, wheat production is often complicated by soil acidification, primarily due to the addition of ammoniacal fertilizers, which can have a strong influence on metal bioavailability, soil chemistry, and microbial communities (Schroder et al., 2011; Lewis et al., 2018). Soil acidification is a global issue currently affecting a large percentage of the world’s arable land (von Uexküll and Mutert, 1995), and the toxicity of soluble Al in acidic soils is thought to be a major factor in limiting plant growth (Foy, 1984). It has been hypothesized that many microbes can produce metal-chelating compounds, such as siderophores and organic acids, that may play a role in metal availability in the soil (Jones et al., 2003; Glick, 2010). Still, it is unclear how fungal plant pathogens respond to Al toxicity at the molecular level. In addition to revealing fundamental molecular mechanisms involved in Al toxicity, understanding how fungal plant pathogens might respond to this important aspect of soil acidification might assist in developing methods of weed seedbank control as soils acidify.

Work examining the *F. avenaceum* genome has shown it is enriched in transcription factors, redox-related proteins, and signal transduction proteins (Lysøe et al., 2014). Additionally, the *F. avenaceum* transcriptome was found to be enriched in gene ontology (GO) categories related to membrane activity, ATP/GTP binding, and calcium ion binding (Lysøe et al., 2014). One objective of the current work is to examine the transcriptomic changes associated with fungal colonization of *A. fatua* caryopses. Doing so should provide key insights into the molecular mechanisms governing *A. fatua* caryopsis colonization. Another objective is to examine the influence of Al toxicity on fungal gene expression in the absence and presence of *A. fatua* caryopses. Addressing this last objective would provide information regarding how *F. avenaceum* responds to aluminum toxicity while also examining how Al influences the fungal transcriptome during colonization of caryopses. The ultimate objective of the work is to elucidate fungal genes which may be of use in future endeavors to develop weed seed suppression technologies through promotion of weed seedbank destruction.

## Materials and Methods

### Fungal Culturing

Filter disk segments containing mycelium from *F. avenaceum* F.a.1 were transferred to potato dextrose agar (PDA: 24 g potato dextrose L^–1^ + 15 g agar L^–1^) plates (25 mL). After 13 days of growth, 6 mm plugs were taken with a sterile core sampler and placed to 25 mL PDA plates that had been amended 72 h earlier, with 1 mL of double-deionized (DDI) sterile H_2_O (PDA-H_2_O), or 1 mL of 10 mM AlCl_3_ (PDA-Al). All plates were incubated in dark conditions at 22°C.

### Plant-Fungal Interaction and Tissue Sampling

**FIGURE 1:**
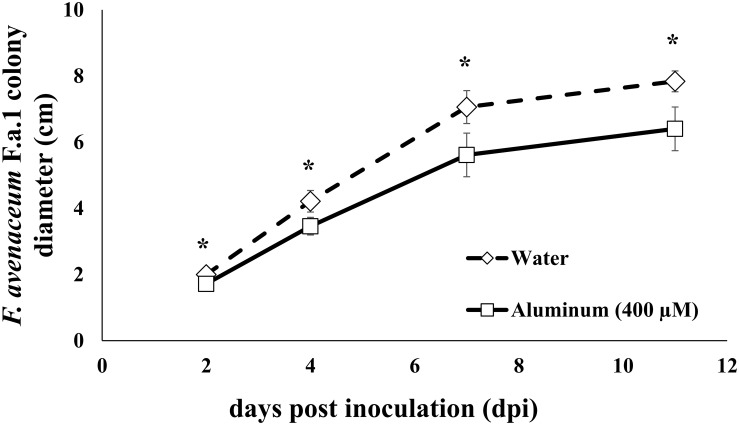
Fungal colony diameter of *Fusarium avenaceum* F.a.1 in cm. Days post inoculation (dpi) is days after transferring a 6 mm plug of colonized potato dextrose agar (PDA; 25 mL) to the center of fresh PDA (25 mL) amended with either 1 mL of water (diamond; dotted line) or 1 mL of 10 mM AlCl_3_ (squares; solid lines; 400 μM is final aluminum concentration). Data represent samples used in transcriptomic studies. Because caryopses were not added until day eight, colony diameter analyses have 12 replicates for each treatment (water or Al). Asterisks indicate statistical significance at alpha = 0.05.

Fungal culture diameter was measured at 2, 4, 7, and 11 days post inoculation (DPI); note that fungal colony diameter data are only shown for samples used in the RNA-Seq studies, so there are 12 replicates per treatment (water and Al) (Figure 1). After 8 days of growth on PDA-H_2_O or PDA-Al, 30 dry wild oat (*Avena fatua*) caryopses were placed along the growing edge of the fungal mycelial mat on nine plates of each treatment. Caryopses were also placed on 25 mL water agar plates (15 g agar L^–1^) with one mL of DDI H_2_O (Agar-H_2_O), or one mL 10 mM AlCl_3_ (Agar-Al), without the fungus; these plates were previously prepared and treated along with the PDA plates described above. After addition of caryopses, the plates were incubated in dark conditions at 15°C for 3 days (72 h). The decreased temperature was used to discourage germination of the caryopses.

After 72 h, caryopses were gently removed from the fungal mat using sterile forceps, large fungal fragments were carefully removed, and then the caryopses were placed in sterile 2 mL tubes. Six replicates per treatment of the caryopses samples were set aside for a separate study, and three replicates of each treatment were used for polyphenol oxidase (PPO) activity assays. The fungal tissue was gently scraped from the PDA plates using a sterile spatula, and placed in sterile 2 mL tubes. All tubes were massed before and after loading samples and were immediately placed in liquid N_2_ after massing. Samples were then maintained at −80°C before RNA extraction. Throughout the manuscript the treatments are indicated as follows, FOW = fungus only + water, FW = fungus + caryopsis + water, FOA = fungus only + Al, and FA = fungus + caryopsis + Al.

### PPO Activity in Caryopses

Whole caryopsis PPO activity was assayed spectrophotometrically as previously described (Fuerst et al., 2018). Using forceps, caryopses were gently removed from Agar-H_2_O and Agar-Al plates, and from the fungal mycelial bed from the fungus grown on PDA-H_2_O and PDA-Al (three replicates each). Three plates per treatment were dedicated to the PPO assays, and three replicates composed of five caryopses each were gathered from each plate and transferred to a tared 2-mL microcentrifuge tube, and samples were re-weighed. Samples were incubated in 1.25 mL substrate solution consisting of 10 mM L-DOPA (L-3,4-dihydroxyphenylalanine) at pH 6.5. Samples were incubated at room temperature on an end-over-end shaker for 25 min and the reaction was terminated with 1 mM tropolone (2-hydroxy-2,4,6-cycloheptatrien-1-one). Samples were centrifuged to remove particulate contaminants and 300 μL of supernatant was transferred to a microtiter plate in duplicate. Absorbance at 475 nm was determined with a spectrophotometer (BioTek Epoch; BioTek Instruments, Inc., Winooski, VT, United States). Results are reported as change in optical density per gram fresh weight of caryopses (gfwt^–1^).

### RNA Extraction, Sequencing, and Analysis

Six replicates from each treatment were used for fungal transcriptome studies. Samples ranging from 56 to 271 mg were collected and put into 2 mL safe-lock tubes (Eppendorf) at −80°C. Samples were prepared by precooling TissueLyser adapter sets in liquid nitrogen and adding two sterilized 2.88 mm stainless steel beads in each sample tube, then sample homogenates were generated using a TissueLyser II (Qiagen) with a frequency setting of 30 for 40 s. About 50 mg sample powder for each sample was collected in 0.5 mL Trizol (Invitrogen, Carlsbad, CA, United States). For RNA extraction, 0.3 mL chloroform was added to 0.5 mL Trizol homogenates, followed by vigorous sample shaking for 2 min. Samples were transferred to 1.5 mL tubes, and then allowed to sit for 3 min at room temperature, followed by centrifugation at 12,000 × *g* for 15 min at 4°C to assist with separation of organic and aqueous phases. The aqueous phase (∼250 μL) was then transferred to a new sterile RNase-free tube and an equal volume of 100 % EtOH was added, with mixing as needed. Samples were further purified using the RNeasy Mini Kit (Qiagen, Valencia, CA, United States) according to the manufacturer’s protocol. The quality and quantity of each RNA sample was assessed using a NanoDrop 2000 Spectrophotometer (Thermo Scientific, Wilmington, DE, United States), and an Agilent 2100 Bioanalyzer (Agilent, Santa Clara, CA, United States).

Libraries were prepared using the TruSeq RNA Library Prep Kit (Illumina, San Diego, CA, United States). Next-generation sequencing was performed by Novogene Inc. using an Illumina NovaSeq 6000 (paired-end, 2 × 150 bp, 20 million reads per sample). Reads were filtered by discarding those with adaptor contamination, uncertain nucleotides >10%, and/or base quality <20 for more than 50% of the read. The *Fusarium avenaceum* genome was used as a reference and mapping was performed using TopHat (v2.0.12) with mismatch = 2 (Trapnell et al., 2009). HTSeq (v0.6.1) was used for quantification using the “union” mode (Anders et al., 2015). DESeq (v1.120.1) was used for assessing differentially expressed genes (DEGs) with significance assessed using an adjusted *p*-value of 0.05 (Anders and Huber, 2010). Reported differentially expressed genes were further trimmed to include only those with |LOG_2_(Fold Change)| ≥2 (which is a 4-fold change), and only genes with at least 14 average reads in one of the treatments being compared were discussed. Genes were annotated using the Swiss-Prot (UniProt Consortium, 2018) and GenBank (Coordinators, 2016) databases (Supplementary Tables S1–S3). Kyoto Encyclopedia of Genes and Genomes (KEGG) (Kanehisa and Goto, 2000) enrichment analysis was performed using *Fusarium graminearum* (Walkowiak et al., 2016) as a reference and KOBAS (v3.0) with significance evaluated at an adjusted *p*-value of 0.05. GO enrichment was assessed using HMMER (v3.1b1) and significance assessed using an adjusted *p*-value of 0.05 (Eddy, 2011). All adjusted *p*-values were obtained using the FDR method. Information regarding RNA quality and RNA-Seq quality control can be found in supplemental information (Supplementary Tables S4–S6). Counts of genes that were uniquely expressed and exhibited statistically significant expression (DEGs) were summarized in Supplementary Table S7; these data were further filtered to only include gene ontology terms with ≥5 DEGs. Raw sequencing data are available via the Sequence Read Archive (SRA; SRA accession: PRJNA595343).

## Results

### Aluminum Influences Colony Formation but Not PPO Activity in Caryopses

**FIGURE 2:**
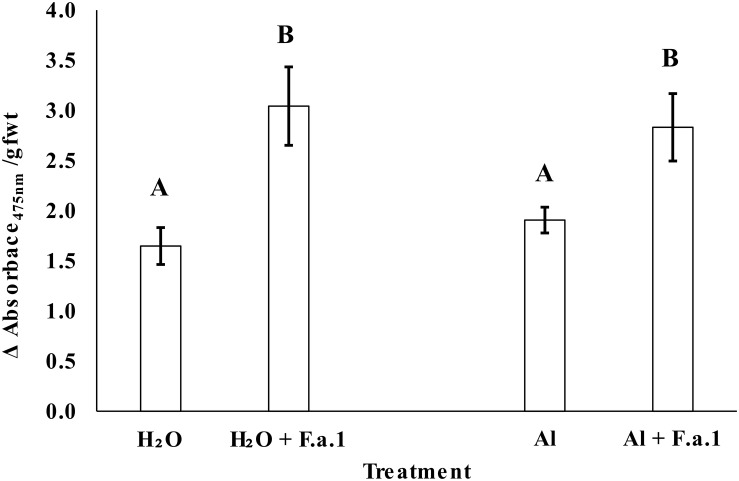
Polyphenol oxidase (PPO) activity in whole caryopses presented as average change (Δ) in absorbance at 475 nm divided by caryopsis grams fresh weight (gfwt). Bars are standard deviation and letters represent significance evaluated at alpha = 0.05. Caryopses in the absence of *Fusarium avenaceum* F.a.1 (F.a.1) were sampled from 25 mL water agar plates amended with 1 mL of water (H_2_O) or 10 mM AlCl_3_ (Al). Caryopses in the presence of F.a.1 were sampled from 25 mL PDA plates with the same amendments.

Chronic sublethal exposure of F.a.1 to 400 μM Al resulted in inhibited fungal colony formation that persisted across the study, starting at 2 days post inoculation (dpi) until tissue harvesting at 11 dpi (Figure 1). Fungal colonies on control plates containing water ultimately reached an average diameter of 7.8 cm, while those exposed to Al reached an average diameter of 6.4 cm. Activity of polyphenol oxidase (PPO) was increased in the caryopses exposed to F.a.1, however, PPO activity was not influenced by addition of Al (Figure 2).

### Fungal Genes Involved in Wild Oat Caryopsis Colonization

**FIGURE 3:**
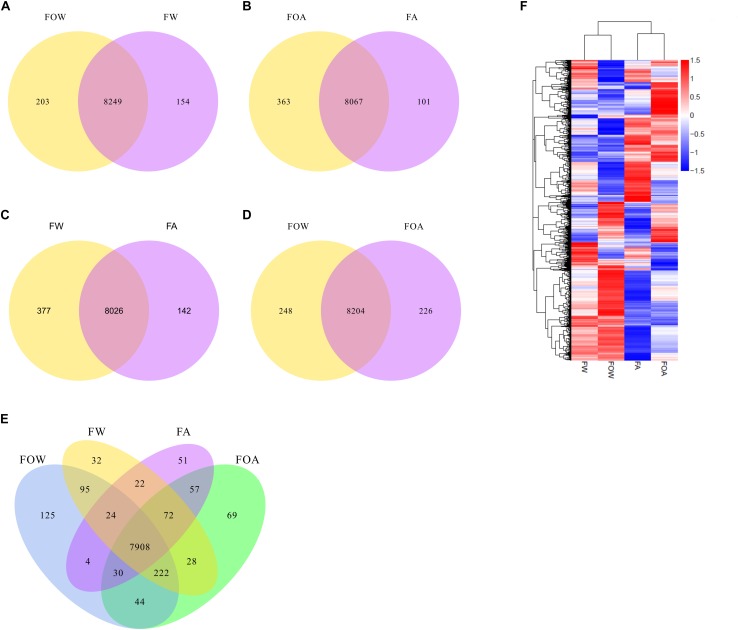
Influence of aluminum exposure and caryopsis colonization on global gene expression. FOW, fungus only exposed to water**;** FW, fungus exposed to water plus caryopses**;** FOA, fungus only exposed to aluminum**;** FA, fungus exposed to aluminum plus caryopses. Co-expression Venn diagrams are shown comparing **(A)** FOW vs. FW, **(B)** FOA vs. FA, **(C)** FW vs. FA **(D)** FOW vs. FOA), and **(E)** all treatments. **(F)** shows a clusters analysis of differentially expressed genes.

In the absence of Al, 8,249 genes were co-expressed in the fungus with (FW) or without the caryopses (FOW). In the presence or absence of the caryopses, 154 and 203 genes were uniquely expressed in the fungus (Figure 3A). Of uniquely expressed genes in the FW treatment, differentially expressed genes were associated with unique gene ontology terms; these terms included oxidation-reduction process, oxidoreductase activity, ion binding, small molecule binding, organic cyclic compound metabolic process, and more (Supplementary Table S7). Induction and repression of genes related to several biological functions were associated with *A. fatua* caryopsis colonization in the FW treatment, including genes involved in virulence/pathogenicity, stress detoxification responses, organic acid metabolism, metal interactions, basic metabolism, and amino acid/peptide/protein metabolism (Tables 1, 2, and Supplementary Table S1). Proteins of many of the induced and repressed genes were localized in various membrane compartments (Tables 1, 2), with several induced uncharacterized transporters being potentially localized to the vacuole membrane (Table 1).

**TABLE 1:** Caryopsis-induced differentially expressed genes in *F. avenaceum* F.a.1. in the absence of aluminum (FW vs. FOW).

Transcript ID	Gene name/function	Log_2_ (Fold change)	Subcellular localization
	**Virulence**		
KIL85362.1	Ga4 desaturase	3.1	NF
KIL90538.1	Ent-kaurene oxidase	3.1	integral component of membrane [GO:0016021]
KIL91977.1	Thaumatin-like protein	2.8	extracellular region [GO:0005576]
KIL89103.1	Catalase-1	2.7	ascospore wall [GO:0005619]; cytosol [GO:0005829]; extracellular region [GO:0005576]
KIL83809.1	Aldehyde dehydrogenase	2.5	cytoplasm [GO:0005737]
KIL87894.1	Oxalate decarboxylase OxdD	2.3	cytoplasm [GO:0005737]
KIL92157.1	Acetyl-CoA hydrolase	2.1	mitochondrion [GO:0005739]
KIL87701.1	Related to 2′-hydroxyisoflavone reductase	2.1	NF
	**Detoxification/Stress**		
KIL92628.1	Uncharacterized MFS-type transporter C409.08	7.1	fungal-type vacuole membrane [GO:0000329]; integral component of plasma membrane [GO:0005887]; plasma membrane [GO:0005886]
KIL88411.1	Aflatoxin B1 aldehyde reductase member 4	6.4	cytosol [GO:0005829]; extracellular exosome [GO:0070062]
KIL94023.1	Peroxisomal catalase	5.2	peroxisome [GO:0005777]
KIL85820.1	Uncharacterized transporter C794.04c	4.3	fungal-type vacuole membrane [GO:0000329]; integral component of plasma membrane [GO:0005887]; plasma membrane [GO:0005886]
KIL87621.1	Nitrosoguanidine resistance protein	3.9	integral component of membrane [GO:0016021]
KIL88862.1	Uncharacterized transporter C36.03c	3.9	endoplasmic reticulum [GO:0005783]; fungal-type vacuole [GO:0000324]; fungal-type vacuole membrane [GO:0000329]; integral component of plasma membrane [GO:0005887]; plasma membrane [GO:0005886]
KIL86787.1	Aldehyde dehydrogenase	3.9	extracellular region [GO:0005576]
KIL88163.1	Cytochrome P450 4F6	3.6	endoplasmic reticulum membrane [GO:0005789]
KIL89486.1	Putative cryptochrome DASH	3.4	NF
KIL90370.1	11-oxo-beta-amyrin 30-oxidase	3.3	integral component of membrane [GO:0016021]
KIL86788.1	Aldehyde dehydrogenase	2.9	NF
KIL89668.1	Glutathione s-transferase	2.8	NF
KIL85730.1	Zinc finger protein MSN4	2.7	cytosol [GO:0005829]; nucleus [GO:0005634]
KIL87533.1	Dienelactone hydrolase	2.6	NF
KIL86609.1	Cytochrome P450 4F5	2.6	endoplasmic reticulum membrane [GO:0005789]
KIL86151.1	Brefeldin A resistance protein	2.6	integral component of membrane [GO:0016021]; plasma membrane [GO:0005886]
KIL93695.1	Isotrichodermin C-15 hydroxylase	2.6	integral component of membrane [GO:0016021]
KIL95553.1	Glutathione-independent glyoxalase HSP31	2.6	cytoplasmic stress granule [GO:0010494]; P-body [GO:0000932]
KIL90371.1	Cholesterol 7-alpha-monooxygenase	2.4	cell [GO:0005623]; endoplasmic reticulum membrane [GO:0005789]; integral component of membrane [GO:0016021]; intracellular membrane-bounded organelle [GO:0043231]
KIL84900.1	Activator of stress genes 1	2.3	nucleus [GO:0005634]
	**Oxidoreduction**		
KIL87958.1	Zinc-type alcohol dehydrogenase-like protein PB24D3.08c	4.7	cytosol [GO:0005829]; nucleus [GO:0005634]
KIL86608.1	NADH-cytochrome b5 reductase 1	4.6	endoplasmic reticulum membrane [GO:0005789]; integral component of membrane [GO:0016021]; mitochondrial outer membrane [GO:0005741]
KIL94114.1	Uncharacterized oxidoreductase TM_0019	2.7	NF
KIL83713.1	Uncharacterized oxidoreductase DltE	2.3	cytoplasm [GO:0005737]
KIL94143.1	FAD dependent oxidoreductase domain-containing protein	2.1	integral component of membrane [GO:0016021]
KIL95101.1	Uncharacterized oxidoreductase C4H3.08	2	cytosol [GO:0005829]; nucleus [GO:0005634]
KIL95944.1	External alternative NAD(P)H-ubiquinone oxidoreductase B1, mitochondrial	2	extrinsic component of mitochondrial inner membrane [GO:0031314]; mitochondrial intermembrane space [GO:0005758]; mitochondrion [GO:0005739]; peroxisome [GO:0005777]
	**Organic Acids**		
KIL86435.1	Malic acid transport protein	2.5	endoplasmic reticulum [GO:0005783]; integral component of membrane [GO:0016021]
KIL87976.1	2-methylisocitrate lyase, mitochondrial	2	mitochondrial matrix [GO:0005759]

*Differentially expressed genes were filtered to those with Log_2_(fold change) ≥2. Subcellular localization is given based on gene ontology. NF = not found.*

**TABLE 2:** Caryopsis-repressed differentially expressed genes in *F. avenaceum* F.a.1. in the absence of aluminum (FW vs. FOW).

Transcript ID	Gene name/function	Log_2_ (Fold change)	Subcellular localization
	**Development**		
KIL95720.1	Protein fluG	−2.2	cytoplasm [GO:0005737]
KIL88384.1	Cell surface protein mas1	−2	NF
	**Transport**		
KIL94396.1	Uncharacterized transporter YIL166C	−3.5	cell periphery [GO:0071944]; fungal-type vacuole [GO:0000324]; integral component of plasma membrane [GO:0005887]; intrinsic component of membrane [GO:0031224]; membrane [GO:0016020]
KIL94330.1	Putative metal chaperone YciC	−3.4	NF
	**Cell wall-related**		
KIL88383.1	Bnr repeat-containing glycosyl hydrolase	−2.9	NF
KIL85999.1	Probable endo-beta-1,4-glucanase D	−2.1	extracellular region [GO:0005576]
	**Virulence**		
KIL88379.1	Secreted protein	−4.4	NF
KIL88377.1	Secreted protein	−4	NF
KIL90234.1	bys1 protein	−2.2	NF

*Differentially expressed genes were filtered to those with Log_2_(fold change) ≤−2. Subcellular localization is given based on gene ontology. NF = not found.*

### Fungal Genes Involved in Wild Oat Caryopsis Colonization During Aluminum Exposure

Compared with the control, Al exposure led to slightly fewer genes (8,067) being co-expressed in the fungus with (FA) or without the caryopses (FOA). In the FA and FOA treatments, 101 and 363 genes were uniquely expressed in the fungus, respectively (Figure 3B). Of uniquely expressed genes in the FA treatment, differentially expressed genes were associated with transporter activity (Supplementary Table S7). In the FOW treatment, 66 gene ontology terms were unique compared with the FW treatment, including primary metabolic process, macromolecule metabolic process, nitrogen compound metabolic process, cellular aromatic compound metabolic process, cellular nitrogen compound metabolic process, and more (Supplementary Table S7).

Genes related to several biological functions were differentially expressed during *A. fatua* caryopsis colonization and Al exposure (FA), including genes involved in iron metabolism, stress/defense responses, basic metabolism, metal-related responses, and amino acid/peptide/protein metabolism, and phosphate-related metabolism (Tables 3, 4 and Supplementary Table S1). While many of the induced and repressed genes were found to encode proteins potentially localized in cellular membranes, several of the induced cell wall-related genes were found to be localized in the extracellular region (Tables 3, 4).

**TABLE 3:** Caryopsis-induced differentially expressed genes in *F. avenaceum* F.a.1, during aluminum exposure (FA vs. FOA).

Transcript ID	Gene name/function	Log_2_ (Fold change)	Subcellular localization
	**Virulence**		
KIL88590.1	Polyketide synthase PksJ	4.3	cytoplasm [GO:0005737]
KIL85362.1	Ga4 desaturase	3.5	NF
KIL84112.1	Fumitremorgin C synthase	3.4	integral component of membrane [GO:0016021]
KIL83610.1	NAD/NADP-dependent betaine aldehyde dehydrogenase	3.3	NF
KIL86378.1	Nonribosomal peptide synthetase 8	3.2	NF
KIL90808.1	Monooxygenase af470	2.9	integral component of membrane [GO:0016021]
KIL86961.1	Phenolic acid decarboxylase padc	2.8	NF
KIL86088.1	O-methylsterigmatocystin oxidoreductase	2.7	NF
KIL85314.1	Copper amine oxidase 1	2.5	NF
KIL95831.1	Global transcription regulator sge1	2.5	nucleus [GO:0005634]
KIL87709.1	Small secreted protein	2.2	NF
KIL89840.1	Acyl-CoA dehydrogenase	2.2	plasma membrane [GO:0005886]
KIL89103.1	Catalase-1	2.1	ascospore wall [GO:0005619]; cytosol [GO:0005829]; extracellular region [GO:0005576]
KIL86331.1	Related to OrfH-unknown, trichothecene gene cluster	2	NF
	**Detoxification/Stress**		
KIL88862.1	Uncharacterized transporter C36.03c	4.3	endoplasmic reticulum [GO:0005783]; fungal-type vacuole [GO:0000324]; fungal-type vacuole membrane [GO:0000329]; integral component of plasma membrane [GO:0005887]; plasma membrane [GO:0005886]
KIL95553.1	Glutathione-independent glyoxalase HSP31	3.9	cytoplasmic stress granule [GO:0010494]; P-body [GO:0000932]
KIL84900.1	Activator of stress genes 1	3.5	nucleus [GO:0005634]
KIL86249.1	Pisatin demethylase	2.8	NF
KIL86788.1	Aldehyde dehydrogenase	2.5	NF
KIL95994.1	Uncharacterized MFS-type transporter C1271.10c	2.5	cell cortex [GO:0005938]; integral component of plasma membrane [GO:0005887]
KIL87621.1	Nitrosoguanidine resistance protein sng1	2.3	integral component of membrane [GO:0016021]
KIL88711.1	Csbd-like domain-containing protein	2.2	NF
KIL85820.1	Uncharacterized transporter C794.04c	2.2	fungal-type vacuole membrane [GO:0000329]; integral component of plasma membrane [GO:0005887]; plasma membrane [GO:0005886]
KIL86151.1	Brefeldin A resistance protein	2.2	integral component of membrane [GO:0016021]; plasma membrane [GO:0005886]
KIL96267.1	Acyl-CoA dehydrogenase family member 10	2	mitochondrion [GO:0005739]
	Siderophore		
KIL86380.1	Nonribosomal peptide synthetase 4 (sidD)	3.8	cytoplasm [GO:0005737]
	**Iron-related**		
KIL88164.1	NADH-cytochrome b5 reductase 1	5.4	endoplasmic reticulum membrane [GO:0005789]; integral component of membrane [GO:0016021]; mitochondrial outer membrane [GO:0005741]
KIL90643.1	Bifunctional P-450:NADPH-P450 reductase	3.7	membrane [GO:0016020]
	**Oxidoreduction**		
KIL87958.1	Zinc-type alcohol dehydrogenase-like protein PB24D3.08c	6	cytosol [GO:0005829]; nucleus [GO:0005634]
KIL94143.1	FAD dependent oxidoreductase domain-containing protein	3.3	integral component of membrane [GO:0016021]
KIL93076.1	Uncharacterized oxidoreductase C736.13	2.8	NF
KIL83713.1	Uncharacterized oxidoreductase DltE	1.9	cytoplasm [GO:0005737]
	**Cell wall-related**		
KIL88359.1	LysM domain-containing protein ARB_05157	3.4	extracellular region [GO:0005576]
KIL88726.1	Glucan endo-1,3-beta-glucosidase A1	3	extracellular region [GO:0005576]
KIL94232.1	Glucan endo-1,3-beta-glucosidase A1	2.7	extracellular region [GO:0005576]
KIL88795.1	Cell wall protein phiA	2.6	cell wall [GO:0005618]; extracellular region [GO:0005576]
KIL86896.1	Pectinesterase	2.4	extracellular region [GO:0005576]
KIL88211.1	Beta-glucosidase	2.3	integral component of membrane [GO:0016021]

*Differentially expressed genes were filtered to those with Log_2_(fold change) ≥2. Subcellular localization is given based on gene ontology. NF = not found.*

**TABLE 4:** Caryopsis-repressed differentially expressed genes in *F. avenaceum* F.a.1, during aluminum exposure (FA vs. FOA).

Transcript ID	Gene name/function	Log_2_(Fold change)	Subcellular localization
	**Cell wall-related**		
KIL84667.1	Putative glycosyl hydrolase	−3.6	integral component of membrane [GO:0016021]
KIL92158.1	Probable glucan endo-1,6-beta-glucosidase B	−2.1	extracellular region [GO:0005576]
KIL84546.1	Related to beta-1,3-glucan binding protein	−2.1	NF
	**Detoxification/Stress**		
KIL85974.1	Acriflavine sensitivity control protein acr-2	−3.3	nucleus [GO:0005634]
KIL87588.1	Aminoglycoside phosphotransferase	−2.6	NF
KIL87830.1	25-hydroxycholesterol 7-alpha-hydroxylase	−2.4	endoplasmic reticulum membrane [GO:0005789]; integral component of membrane [GO:0016021]
KIL90821.1	Dimethylaniline monooxygenase [N-oxide-forming] 2	−2.3	endoplasmic reticulum membrane [GO:0005789]; integral component of membrane [GO:0016021]; membrane [GO:0016020]
KIL94286.1	Drug resistance protein YOR378W	−2.3	cell periphery [GO:0071944]; integral component of plasma membrane [GO:0005887]; plasma membrane [GO:0005886]
KIL94224.1	Putative HC-toxin efflux carrier TOXA	−2.2	integral component of membrane [GO:0016021]; integral component of plasma membrane [GO:0005887]
KIL85529.1	Capreomycidine synthase	−2.1	NF
	**Development**		
KIL84622.1	Sphingoid long-chain base transporter RSB1	−4.5	integral component of membrane [GO:0016021]; plasma membrane [GO:0005886]
KIL92179.1	UNC93-like protein C922.05c	−2.7	cytoplasm [GO:0005737]; integral component of plasma membrane [GO:0005887]
	Transcriptional regulation		
KIL86550.1	Transcription factor	−2.1	nucleus [GO:0005634]
	**Virulence**		
KIL88216.1	Probable sterigmatocystin biosynthesis P450 monooxygenase STCB	−2.6	NF
KIL94112.1	Subtilisin-like protease 3	−2.6	endoplasmic reticulum [GO:0005783]; extracellular space [GO:0005615]; fungal-type vacuole lumen [GO:0000328]
KIL91080.1	Alcohol dehydrogenase 3, mitochondrial	−2.1	mitochondrial matrix [GO:0005759]

*Differentially expressed genes were filtered to those with Log_2_(fold change) ≥2. Subcellular localization is given based on gene ontology. NF = not found.*

### The Influence of Al on Caryopsis Colonization

Caryopsis colonization resulted in co-expression of 8,026 genes in the fungus exposed to water (FW) or Al (FA). Additionally, 142 and 377 genes were uniquely expressed in the fungus in the presence or absence of Al, respectively (Figure 3C). Compared with the FW treatment, uniquely expressed DEGs were associated with three unique gene ontology terms in the FA treatment, including cofactor binding, coenzyme binding, and ion transport (Supplementary Table S7).

Induction of genes related to several biological functions were associated with Al exposure during *A. fatua* caryopsis colonization (FA) when compared to the FW treatment, including genes involved in siderophore metabolism, iron metabolism, stress/defense responses, drug resistance, basic metabolism, metal-related responses, and phosphate-related metabolism (Tables 5, 6 and Supplementary Table S1). Proteins of both Al-repressed and Al-induced genes were found to be potentially localized to membranes and the cytosol/cytoplasm (Tables 5, 6). Two genes associated with transport and detoxification (uncharacterized membrane protein YJR124C and leptomycin B resistance protein pmd1, respectively), were found to be induced by Al, with the associated proteins potentially being localized in the vacuole membrane (Table 5). Laccase-2 was found to be repressed by Al in the FA treatment compared to the FW treatment, and was found to be potentially partitioned to the extracellular region (Table 6).

**TABLE 5:** Aluminum-induced differentially expressed genes in *F. avenaceum* F.a.1 during caryopsis colonization (FA vs. FW).

Transcript ID	Gene name/function	Log_2_(Fold change)	Subcellular localization
	**Basic metabolism**		
KIL85664.1	Cytochrome b2, mitochondrial	4.3	mitochondrial intermembrane space [GO:0005758]; respirasome [GO:0070469]
KIL88415.1	D-xylose 1-dehydrogenase [NADP(+)] 2	3.6	extracellular region [GO:0005576]
KIL88271.1	Glucokinase	3.2	cell [GO:0005623]
KIL94987.1	Uncharacterized methyltransferase C25B8.09	3.1	cytosol [GO:0005829]; nucleus [GO:0005634]
KIL86846.1	Alkali-sensitive linkage protein 1	3	endoplasmic reticulum [GO:0005783]; external side of cell wall [GO:0010339]; extracellular region [GO:0005576]; fungal-type cell wall [GO:0009277]; Golgi apparatus [GO:0005794]
KIL93784.1	Cytoplasmic 60S subunit biogenesis factor REI1 homolog	2.8	cytoplasm [GO:0005737]
KIL87652.1	NADH oxidase	2.7	NF
KIL87020.1	Enoyl-CoA hydratase domain-containing protein 2, mitochondrial	2.5	mitochondrion [GO:0005739]
KIL85122.1	putative mnn4-regulates the mannosylphosphorylation	2.2	integral component of membrane [GO:0016021]
KIL94407.1	Uncharacterized CDP-alcohol phosphatidyltransferase class-I family protein C22A12.08c	2.1	membrane [GO:0016020]; mitochondrion [GO:0005739]
KIL94408.1	Glycerol 2-dehydrogenase [NADP(+)]	2.1	NF
KIL89558.1	Glucose-repressible gene protein	2	NF
KIL89806.1	Phosphatidate phosphatase APP1	2	actin cortical patch [GO:0030479]
	**Cell wall-related**		
KIL87303.1	Endopolygalacturonase AN8327	3.9	extracellular region [GO:0005576]
KIL88211.1	Beta-glucosidase	2.5	integral component of membrane [GO:0016021]
KIL94677.1	Endochitinase B1	2.3	extracellular region [GO:0005576]
	**Detoxification/Stress**		
KIL93713.1	Multidrug resistance-associated protein 1	8.4	basolateral plasma membrane [GO:0016323]; integral component of membrane [GO:0016021]; membrane [GO:0016020]
KIL86332.1	Putative cytochrome P450 CYP13A7	4	NF
KIL88897.1	Leptomycin B resistance protein pmd1	3.8	fungal-type vacuole [GO:0000324]; integral component of membrane [GO:0016021]; plasma membrane [GO:0005886]
KIL91637.1	Multidrug resistance protein 2	2.8	integral component of membrane [GO:0016021]
KIL88089.1	4-sulfomuconolactone hydrolase	2.3	NF
	**Development**		
KIL86107.1	Dimethylaniline monooxygenase [N-oxide-forming] 5	4.7	NF
KIL90244.1	Infection structure specific protein	2.9	NF
KIL90075.1	Uncharacterized FAD-linked oxidoreductase YvdP	2.2	spore wall [GO:0031160]
	**Oxidoreduction**		
KIL87669.1	Uncharacterized FAD-linked oxidoreductase ARB_02478	4.3	extracellular region [GO:0005576]
KIL95747.1	Dimethyl-sulfide monooxygenase	3.6	NF
KIL87414.1	Isoamyl alcohol	2.5	NF
KIL88501.1	Uncharacterized FAD-linked oxidoreductase ARB_02478	2	extracellular region [GO:0005576]
	**Transport**		
KIL86435.1	Malic acid transport protein	3.3	endoplasmic reticulum [GO:0005783]; integral component of membrane [GO:0016021]
KIL86675.1	Uncharacterized transporter PB1C11.03	3	endoplasmic reticulum [GO:0005783]; integral component of plasma membrane [GO:0005887]; intrinsic component of membrane [GO:0031224]
KIL85821.1	Uncharacterized membrane protein YJR124C	2.9	fungal-type vacuole membrane [GO:0000329]; integral component of plasma membrane [GO:0005887]
KIL91580.1	Iron transport multicopper oxidase FET3	2.8	cell [GO:0005623]; high-affinity iron permease complex [GO:0033573]
KIL93709.1	Protein kes1	2.5	cell division site [GO:0032153]; cytosol [GO:0005829]; intracellular membrane-bounded organelle [GO:0043231]; membrane [GO:0016020]
KIL93707.1	P-type cation-transporting ATPase	2.3	cell [GO:0005623]; integral component of plasma membrane [GO:0005887]; plasma membrane [GO:0005886]; proteasome core complex [GO:0005839]
KIL87110.1	Hexose transporter 2	2	integral component of membrane [GO:0016021]
	**Virulence**		
KIL88898.1	Apoptosis-inducing factor 2	8.6	cytoplasm [GO:0005737]; cytosol [GO:0005829]; integral component of membrane [GO:0016021]; lipid droplet [GO:0005811]; mitochondrial outer membrane [GO:0005741]; mitochondrion [GO:0005739]
KIL93640.1	L-ornithine N(5)-monooxygenase (Siderophore Biosynthesis)	6.3	NF
KIL94193.1	Apoptosis-inducing factor 2	4.9	integral component of membrane [GO:0016021]; mitochondrial outer membrane [GO:0005741]
KIL84112.1	Fumitremorgin C synthase	4.7	integral component of membrane [GO:0016021]
KIL84938.1	Endoglucanase-7	3.9	extracellular region [GO:0005576]
KIL93826.1	Aspergillopepsin-2	2.8	NF
KIL93932.1	Related to acetylxylan esterase	2.5	NF
KIL85313.1	Aldehyde dehydrogenase	2.5	cytoplasm [GO:0005737]
KIL84075.1	Peroxiredoxin-1	2.3	cell [GO:0005623]; cytosol [GO:0005829]; nucleus [GO:0005634]

*Differentially expressed genes were filtered to those with Log_2_(fold change) ≥2. Subcellular localization is given based on gene ontology. NF = not found.*

**TABLE 6:** Aluminum-repressed differentially expressed genes in *F. avenaceum* F.a.1 during caryopsis colonization (FA vs. FW).

Transcript ID	Gene name/function	Log_2_(Fold change)	Subcellular localization
	**Basic metabolism**		
KIL90295.1	Probable quinate permease	−6	integral component of plasma membrane [GO:0005887]
KIL94775.1	Fibronectin type III domain protein	−2.4	NF
KIL95538.1	Protein SERAC1	−2.3	endoplasmic reticulum [GO:0005783]; extracellular matrix [GO:0031012]; integral component of membrane [GO:0016021]; mitochondria-associated endoplasmic reticulum membrane [GO:0044233]; mitochondrion [GO:0005739]
KIL86771.1	alpha beta-hydrolase	−2.1	NF
KIL89429.1	Uncharacterized PH domain-containing protein YPR091C	−2	cell periphery [GO:0071944]; endoplasmic reticulum [GO:0005783]; endoplasmic reticulum membrane [GO:0005789]; integral component of membrane [GO:0016021]; nucleus-vacuole junction [GO:0071561]
KIL90108.1	Alpha-glucosidase	−2	cytosol [GO:0005829]; nucleus [GO:0005634]
	**Detoxification/Stress**		
KIL89371.1	Ent-kaurene oxidase	−8.5	integral component of membrane [GO:0016021]
KIL88204.1	Phenol 2-monooxygenase	−7	NF
KIL84575.1	Peroxisomal catalase	−4.2	fungal-type cell wall [GO:0009277]; peroxisome [GO:0005777]
KIL88419.1	Glutathione reductase	−2.9	cell [GO:0005623]; cytosol [GO:0005829]; mitochondrion [GO:0005739]; nucleus [GO:0005634]
KIL94286.1	Drug resistance protein YOR378W	−2.6	cell periphery [GO:0071944]; integral component of plasma membrane [GO:0005887]; plasma membrane [GO:0005886]
KIL87830.1	25-hydroxycholesterol 7-alpha-hydroxylase	−2.5	endoplasmic reticulum membrane [GO:0005789]; integral component of membrane [GO:0016021]
KIL93328.1	DNA damage response protein kinase DUN1	−2.3	cytoplasm [GO:0005737]; nucleus [GO:0005634]
KIL83874.1	Probable nitronate monooxygenase	−2.1	NF
KIL84602.1	HET-domain-containing protein	−2	NF
KIL93910.1	Cytochrome P450 1A1	−2	endoplasmic reticulum membrane [GO:0005789]
	**Development**		
KIL88563.1	Vegetative incompatibility protein HET-E-1	−3	NF
KIL90704.1	Vegetative incompatibility protein HET-E-1	−2.7	NF
KIL88384.1	cell surface protein mas1 [Fusarium langsethiae]	−2.7	NF
KIL90265.1	Vegetative incompatibility protein HET-E-1	−2.6	NF
KIL92404.1	Vegetative incompatibility protein HET-E-1	−2.2	NF
KIL86170.1	Vegetative incompatibility protein HET-E-1	−2.1	NF
KIL83903.1	Vegetative incompatibility protein HET-E-1	−2	NF
	**Nitrate assimilation**		
KIL84574.1	Sulfite oxidase, mitochondrial	−3.8	mitochondrial intermembrane space [GO:0005758]; mitochondrial matrix [GO:0005759]; mitochondrion [GO:0005739]
	**Oxidoreduction**		
KIL89100.1	UDP-N-acetyl-D-glucosamine 6-dehydrogenase	−2.4	integral component of membrane [GO:0016021]
	**Transcriptional regulation**		
KIL90031.1	Transcription factor	−2	nucleus [GO:0005634]
	**Transport**		
KIL85428.1	Bypass of stop codon protein 6	−2.4	Golgi apparatus [GO:0005794]; integral component of plasma membrane [GO:0005887]; membrane [GO:0016020]
KIL89987.1	Uncharacterized ABC transporter ATP-binding protein/permease YOL075C	−2.3	cell periphery [GO:0071944]; fungal-type vacuole membrane [GO:0000329]; integral component of membrane [GO:0016021]
KIL94326.1	Vacuolar iron transporter 1.2	−2.3	NF
KIL90349.1	Sodium/potassium-transporting ATPase subunit alpha	−2.1	integral component of membrane [GO:0016021]; plasma membrane [GO:0005886]
KIL84309.1	Probable inactive 1-aminocyclopropane-1-carboxylate synthase-like protein 2	−2.1	NF
KIL91916.1	Uncharacterized transporter C3H1.06c	−2	endoplasmic reticulum [GO:0005783]; integral component of membrane [GO:0016021]; integral component of plasma membrane [GO:0005887]
	**Virulence**		
KIL86397.1	Conidial yellow pigment biosynthesis polyketide synthase	−4.4	NF
KIL87828.1	Nonribosomal peptide synthetase 8	−2.7	NF
KIL89330.1	Versicolorin B synthase	−2.7	cytosol [GO:0005829]
KIL89340.1	Fumitremorgin C synthase	−2.3	cytoplasm [GO:0005737]; integral component of membrane [GO:0016021]; intracellular membrane-bounded organelle [GO:0043231]
KIL93322.1	Laccase-2	−2	extracellular region [GO:0005576]

*Differentially expressed genes were filtered to those with Log_2_(Fold Change) ≤−2. Subcellular localization is given based on gene ontology. NF = not found.*

### Fungal Transcriptomic Responses to Aluminum Exposure

Exposure of F.a.1 to Al (FOA) or water (FOW) resulted in the co-expression of 8,204 genes in the absence of caryopses (Figure 3D). In the FOW treatment, 248 genes were uniquely expressed, while 226 genes were uniquely expressed during Al exposure (FOA). Genes uniquely expressed in the FOA treatment compared with FOW, were associated with 33 unique GO terms, including several protein-related GO terms, ion transport, organic substance transport, organonitrogen compound metabolic process, organic acid transport, and more (Supplementary Table S7). Compared with the FOA treatment, the FOW treatment was associated with 22 unique GO terms, including several related to nucleic acid metabolism, carbohydrate derivative binding, lipid biosynthetic processes, ATP binding, iron ion binding, oxidoreductase activity (acting on CH-OH group donors, and more) (Supplementary Table S7).

Additionally, Al exposure led to induction of several genes associated with siderophore transport, iron metabolism, organic acid metabolism, and metals, as well as, genes associated with stress/defense, and drug resistance (Tables 7–9 and Supplementary Table S1). Proteins of most of the induced siderophore-related genes were found to be potentially localized to various membranes throughout the cell (Tables 7, 8). Siderophore iron transporter 3 was found to potentially be localized to cell, cytoplasm, integral component of membrane, integral component of plasma membrane, and/or plasma membrane (Table 7). Siderophore iron transporter 1 was found to be potentially localized to any of several compartments, including cell, cytoplasmic vesicle, endosome, endosome membrane, fungal-type vacuole, integral component of plasma membrane, plasma membrane, and/or vacuolar membrane (Table 7). Several Al-induced virulence and detoxification genes were found to potentially encode for proteins that might partition to the extracellular space, including acetyl-coenzyme A synthetase, laccase ARB_05828, subtilisin-like protease 3, aflatoxin B1 aldehyde reductase member 4, and aldehyde dehydrogenase (Table 8).

**TABLE 7:** Aluminum-induced differentially expressed genes in *F. avenaceum* F.a.1, in the absence of caryopsis colonization (FOA vs. FOW).

Transcript ID	Gene name/function	Log_2_(Fold change)	Subcellular localization
	**Siderophore-related**		
KIL93715.1	Nonribosomal peptide synthetase 2	9.1	cell [GO:0005623]; cytoplasm [GO:0005737]
KIL94141.1	Siderophore iron transporter mirB	8.8	cell [GO:0005623]; integral component of plasma membrane [GO:0005887]
KIL85942.1	Siderophore iron transporter mirB	7.8	cell [GO:0005623]; integral component of plasma membrane [GO:0005887]
KIL93640.1	L-ornithine N(5)-monooxygenase	6.7	NF
KIL93714.1	Putative lysine N-acyltransferase C17G9.06c	6.2	cytosol [GO:0005829]; nucleus [GO:0005634]
KIL87671.1	Nonribosomal peptide synthetase 4	6	NF
KIL95063.1	Siderophore iron transporter mirB	5.9	cell [GO:0005623]; integral component of plasma membrane [GO:0005887]
KIL87674.1	Siderophore iron transporter mirB	5.8	cell [GO:0005623]; integral component of plasma membrane [GO:0005887]
KIL87673.1	Putative lysine N-acyltransferase C17G9.06c	5.1	cytosol [GO:0005829]; nucleus [GO:0005634]
KIL93712.1	Siderophore iron transporter 3	4.3	cell [GO:0005623]; cytoplasm [GO:0005737]; integral component of membrane [GO:0016021]; integral component of plasma membrane [GO:0005887]; plasma membrane [GO:0005886]
KIL92195.1	Siderophore iron transporter mirA	3.9	integral component of plasma membrane [GO:0005887]
KIL85295.1	Siderophore iron transporter 1	3.4	cell [GO:0005623]; cytoplasmic vesicle [GO:0031410]; endosome [GO:0005768]; endosome membrane [GO:0010008]; fungal-type vacuole [GO:0000324]; integral component of plasma membrane [GO:0005887]; plasma membrane [GO:0005886]; vacuolar membrane [GO:0005774]
KIL88899.1	Putative lysine N-acyltransferase C17G9.06c	2.6	cytosol [GO:0005829]; nucleus [GO:0005634]
KIL93180.1	Siderophore biosynthesis	2.2	NF
	**Iron-related**		
KIL87675.1	Ferri-bacillibactin esterase BesA	6.1	cytoplasm [GO:0005737]
KIL94023.1	Peroxisomal catalase	5	peroxisome [GO:0005777]
KIL94226.1	1,3-propanediol dehydrogenase	3.5	NF
KIL91581.1	Plasma membrane iron permease	2.8	high-affinity iron permease complex [GO:0033573]; plasma membrane [GO:0005886]
KIL93711.1	Ferric reductase transmembrane component 5	2.3	cell [GO:0005623]; integral component of membrane [GO:0016021]; mitochondrion [GO:0005739]; plasma membrane [GO:0005886]
KIL86612.1	Plasma membrane iron permease	2.3	cell [GO:0005623]; endoplasmic reticulum [GO:0005783]; Golgi apparatus [GO:0005794]; high-affinity iron permease complex [GO:0033573]; plasma membrane [GO:0005886]
KIL86611.1	Iron transport multicopper oxidase FET3	2.3	cell [GO:0005623]; endoplasmic reticulum [GO:0005783]; fungal-type vacuole [GO:0000324]; high-affinity iron permease complex [GO:0033573]; plasma membrane [GO:0005886]
	**Organic acid-related**		
KIL86435.1	Malic acid transport protein	4	endoplasmic reticulum [GO:0005783]; integral component of membrane [GO:0016021]
KIL87894.1	Oxalate decarboxylase OxdD	3.7	cytoplasm [GO:0005737]
KIL96152.1	Isocitrate lyase	2.3	glyoxysome [GO:0009514]
KIL87976.1	2-methylisocitrate lyase, mitochondrial	2.3	mitochondrial matrix [GO:0005759]
KIL84732.1	Mitochondrial oxaloacetate transport protein	2	integral component of membrane [GO:0016021]; mitochondrial inner membrane [GO:0005743]; mitochondrion [GO:0005739]

*Differentially expressed genes were filtered to those with Log_2_(Fold Change) ≥2. Subcellular localization is given based on gene ontology. NF = not found.*

**TABLE 8:** Additional aluminum-induced differentially expressed genes in *F. avenaceum* F.a.1, in the absence of caryopsis colonization (FOA vs. FOW).

Transcript ID	Gene name/function	Log_2_(Fold change)	Subcellular localization
	**Virulence**		
KIL88898.1	Apoptosis-inducing factor 2	8.4	cytoplasm [GO:0005737]; cytosol [GO:0005829]; integral component of membrane [GO:0016021]; lipid droplet [GO:0005811]; mitochondrial outer membrane [GO:0005741]; mitochondrion [GO:0005739]
KIL94193.1	Apoptosis-inducing factor 2	6.4	integral component of membrane [GO:0016021]; mitochondrial outer membrane [GO:0005741]
KIL85313.1	Aldehyde dehydrogenase	4.6	cytoplasm [GO:0005737]
KIL94858.1	Acetyl-coenzyme A synthetase	3.3	extracellular region [GO:0005576]
KIL88588.1	Bys1 protein	3.3	NF
KIL88216.1	Probable sterigmatocystin biosynthesis P450 monooxygenase STCB	3	NF
KIL86239.1	Nonribosomal peptide synthetase 1	2.9	cytoplasm [GO:0005737]
KIL93826.1	Aspergillopepsin-2	2.9	NF
KIL90137.1	Related to cornifin B	2.9	NF
KIL87701.1	Related to 2′-hydroxyisoflavone reductase	2.8	NF
KIL85408.1	Alcohol dehydrogenase [NADP(+)]	2.8	apical plasma membrane [GO:0016324]; cytosol [GO:0005829]; synapse [GO:0045202]
KIL84075.1	Peroxiredoxin-1	2.6	cell [GO:0005623]; cytosol [GO:0005829]; nucleus [GO:0005634]
KIL88365.1	Phosphatidylglycerol lysyltransferase	2.6	NF
KIL84112.1	Fumitremorgin C synthase	2.5	integral component of membrane [GO:0016021]
KIL92157.1	Acetyl-CoA hydrolase	2.5	mitochondrion [GO:0005739]
KIL95012.1	Related to secretory lipase	2.5	NF
KIL87715.1	N-acyl homoserine lactonase attm	2.5	NF
KIL86939.1	Necrosis-inducing protein	2.5	NF
KIL94006.1	Laccase ARB_05828	2.4	extracellular region [GO:0005576]
KIL93932.1	Related to acetylxylan esterase	2.4	NF
KIL94112.1	Subtilisin-like protease 3	2.3	endoplasmic reticulum [GO:0005783]; extracellular space [GO:0005615]; fungal-type vacuole lumen [GO:0000328]
KIL96580.1	Infection structure specific protein	2.2	NF
KIL89564.1	Putative aldehyde dehydrogenase-like protein YHR039C	2.1	endoplasmic reticulum [GO:0005783]
KIL92119.1	Related to cornifin B	2.1	NF
KIL84683.1	RecName: Full = Loline biosynthesis cluster 1 transcription factor lolU1	2.1	nucleus [GO:0005634]
KIL92927.1	Putative branched-chain-amino-acid aminotransferase TOXF	2	NF
	**Detoxification/Stress**		
KIL92628.1	Uncharacterized MFS-type transporter C409.08	7.9	fungal-type vacuole membrane [GO:0000329]; integral component of plasma membrane [GO:0005887]; plasma membrane [GO:0005886]
KIL93713.1	Multidrug resistance-associated protein 1	7.6	basolateral plasma membrane [GO:0016323]; integral component of membrane [GO:0016021]; membrane [GO:0016020]
KIL88411.1	Aflatoxin B1 aldehyde reductase member 4	7	cytosol [GO:0005829]; extracellular exosome [GO:0070062]
KIL90653.1	Probable formaldehyde dehydrogenase AdhA	5.6	NF
KIL85974.1	Acriflavine sensitivity control protein acr-2	4.6	nucleus [GO:0005634]
KIL88897.1	Leptomycin B resistance protein pmd1	4.4	fungal-type vacuole [GO:0000324]; integral component of membrane [GO:0016021]; plasma membrane [GO:0005886]
KIL86332.1	Putative cytochrome P450 CYP13A7	4	NF
KIL93695.1	Isotrichodermin C-15 hydroxylase	3.9	integral component of membrane [GO:0016021]
KIL84891.1	NADH-cytochrome b5 reductase 2	3.8	integral component of mitochondrial outer membrane [GO:0031307]; mitochondrial intermembrane space [GO:0005758]
KIL87621.1	Nitrosoguanidine resistance protein sng1	3.4	integral component of membrane [GO:0016021]
KIL88089.1	4-sulfomuconolactone hydrolase	3.3	NF
KIL91637.1	Multidrug resistance protein 2	3.3	integral component of membrane [GO:0016021]
KIL86787.1	Aldehyde dehydrogenase	3	extracellular region [GO:0005576]
KIL94192.1	Ethyl tert-butyl ether degradation ethd	2.5	NF
KIL87482.1	Pyrethroid hydrolase	2.4	NF
KIL84681.1	Putative HC-toxin efflux carrier TOXA	2.3	integral component of membrane [GO:0016021]; integral component of plasma membrane [GO:0005887]
KIL94406.1	(S)-2-haloacid dehalogenase H-109	2.2	NF
KIL84854.1	Tetracycline resistance protein from transposon Tn4351/Tn4400	2.2	cytoplasm [GO:0005737]
KIL87757.1	Nitrosoguanidine resistance protein	2.2	integral component of membrane [GO:0016021]
KIL92588.1	Quinidine resistance protein 2	2.1	cell periphery [GO:0071944]; integral component of plasma membrane [GO:0005887]; plasma membrane [GO:0005886]
KIL88169.1	Glutathione S-transferase PM239X14	2.1	cytosol [GO:0005829]
KIL96198.1	Puromycin N-acetyltransferase	2.1	NF
KIL86804.1	Putative duf636 domain protein	2.1	NF
KIL95076.1	Heat shock protein 16	2.1	cytoplasm [GO:0005737]; cytosol [GO:0005829]; nucleus [GO:0005634]
KIL94213.1	Multidrug resistance-associated protein 1	2	basolateral plasma membrane [GO:0016323]; integral component of membrane [GO:0016021]; membrane [GO:0016020]
KIL93503.1	Putative HC-toxin efflux carrier TOXA	2	integral component of membrane [GO:0016021]; integral component of plasma membrane [GO:0005887]

*Differentially expressed genes were filtered to those with Log_2_(Fold Change) ≥2. Subcellular localization is given based on gene ontology. NF = not found.*

**TABLE 9:** Aluminum-repressed differentially expressed genes in *F. avenaceum* F.a.1 in the absence of caryopsis colonization (FOA vs. FOW).

Transcript ID	Gene name/function	Log_2_(Fold change)	Subcellular localization
	**Iron-related**		
KIL86453.1	Cytochrome P450 52A13	−5	membrane [GO:0016020]
KIL95759.1	Psi-producing oxygenase A	−3.6	NF
KIL94326.1	Vacuolar iron transporter 1.2	−3.6	cell [GO:0005623]; integral component of membrane [GO:0016021]; vacuolar membrane [GO:0005774]
KIL88199.1	Ferric/cupric reductase transmembrane component 2	−2.7	cell [GO:0005623]; fungal-type vacuole [GO:0000324]; integral component of membrane [GO:0016021]; plasma membrane [GO:0005886]
KIL87695.1	Probable deferrochelatase/peroxidase YfeX	−2.5	cytoplasm [GO:0005737]; cytosol [GO:0005829]
	**Virulence**		
KIL88590.1	Polyketide synthase PksJ	−5.1	cytoplasm [GO:0005737]
KIL84575.1	Peroxisomal catalase	−4.8	fungal-type cell wall [GO:0009277]; peroxisome [GO:0005777]
KIL95888.1	Manganese peroxidase 2	−4.2	extracellular region [GO:0005576]
KIL89374.1	Probable polyketide synthase 1	−3.9	NF
KIL93997.1	Protein SnodProt1	−3.3	extracellular region [GO:0005576]
KIL85985.1	Alcohol dehydrogenase 1	−3.2	cytoplasm [GO:0005737]
KIL89373.1	Transcription factor MYB98	−2.9	nucleus [GO:0005634]
KIL89340.1	Fumitremorgin C synthase	−2.5	cytoplasm [GO:0005737]; integral component of membrane [GO:0016021]; intracellular membrane-bounded organelle [GO:0043231]
KIL93322.1	Laccase-2	−2.2	extracellular region [GO:0005576]
KIL93994.1	Oxalate decarboxylase OxdC (Organic Acid)	−2.2	cytoplasm [GO:0005737]
KIL95972.1	Acyl-coenzyme A:6-aminopenicillanic-acid-acyltransferase 40 kDa form	−2.2	NF
KIL90502.1	Aldehyde dehydrogenase	−2.1	cytoplasm [GO:0005737]
KIL87641.1	6-hydroxynicotinate 3-monooxygenase	−2.1	NF
	**Detoxification/Stress**		
KIL86447.1	Cytochrome P450 52A11	−4.1	membrane [GO:0016020]
KIL88204.1	Phenol 2-monooxygenase	−3.9	NF
KIL89371.1	Ent-kaurene oxidase	−3.8	integral component of membrane [GO:0016021]
KIL88419.1	Glutathione reductase	−2.7	cell [GO:0005623]; cytosol [GO:0005829]; mitochondrion [GO:0005739]; nucleus [GO:0005634]
KIL87166.1	Disulfide-bond oxidoreductase YfcG	−2.5	NF
KIL87179.1	Putative dioxygenase subunit alpha YeaW	−2.1	NF
KIL95756.1	Apoptosis-inducing factor 1	−2	cytosol [GO:0005829]; mitochondrial inner membrane [GO:0005743]; mitochondrial outer membrane [GO:0005741]; nucleus [GO:0005634]
KIL89827.1	Putative glutathione-dependent formaldehyde-activating enzyme	−2	NF

*Differentially expressed genes were filtered to those with Log_2_(Fold Change) ≤−2. Subcellular localization is given based on gene ontology. NF = not found.*

## Gene Ontology (GO) Enrichment and KEGG (Kyoto Encyclopedia of Genes and Genomes) Pathway Analysis

### Chronic Aluminum Exposure Influences Gene Ontology Enrichment

Chronic Exposure of F.a.1 to sublethal concentrations of Al (FOA) was associated with changes in the enrichment of many genes from ontology groups associated with biological and molecular processes. Aluminum exposure was related to induction of genes involved in biological and metabolic processes, catalytic activity, single-organism process, single-organism metabolic process, oxidoreductase activity, oxidation-reduction process, organonitrogen compound metabolic process, small molecule metabolic process, organonitrogen compound biosynthetic process, carboxylic acid metabolic process, oxoacid metabolic process, and organic acid metabolic process (Table 10). Aluminum exposure was also related to the repression of genes associated with gene ontology groups, including biological regulation, cation binding, regulation of cellular process, regulation of biological process, metal ion binding, transition metal ion binding, zinc ion binding, nucleic acid binding transcription factor activity, transcription factor activity, and sequence-specific DNA binding (Table 10). Repression was also observed of several iron-related genes in response to Al exposure. KEGG pathway analysis showed Al exposure led to induction of pathways associated with biosynthesis of secondary metabolites, biosynthesis of amino acids, carbon metabolism, 2-oxocarboxylic acid metabolism, cysteine and methionine metabolism, propanoate metabolism, lysine biosynthesis, and, valine, leucine and isoleucine degradation (Table 11).

**TABLE 10:** Enriched and depleted gene ontology terms associated with Al exposure in the absence of the caryopses (FOA).

Enriched		

Term Type	Description	DEG
Biological process	Biological process	860
Biological process	Metabolic process	686
Molecular function	Catalytic activity	658
Biological process	Single-organism process	530
Biological process	Single-organism metabolic process	362
Molecular function	Oxidoreductase activity	221
Biological process	Oxidation-reduction process	218
Biological process	Organonitrogen compound metabolic process	152
Biological process	Small molecule metabolic process	128
Biological process	Single-organism biosynthetic process	121
Biological process	Organonitrogen compound biosynthetic process	102
Biological process	Carboxylic acid metabolic process	86
Biological process	Oxoacid metabolic process	86
Biological process	Organic acid metabolic process	86
Biological process	Cellular amino acid metabolic process	58
Molecular function	Lyase activity	56
Molecular function	Transferase activity, transferring acyl groups	51
Biological process	Small molecule biosynthetic process	47
Biological process	Alpha-amino acid metabolic process	37
Biological process	Organic acid biosynthetic process	35
Biological process	Carboxylic acid biosynthetic process	35
Molecular function	Active transmembrane transporter activity	26
Molecular function	Hydrolase activity, acting on acid anhydrides, catalyzing transmembrane movement of substances	25
Molecular function	Primary active transmembrane transporter activity	23
Molecular function	P-P-bond-hydrolysis-driven transmembrane transporter activity	23
Molecular function	Pyridoxal phosphate binding	23
Molecular function	ATPase activity, coupled to transmembrane movement of substances	22
Molecular function	ATPase activity, coupled to movement of substances	22
Molecular function	ATPase activity, coupled	22
Molecular function	Carbon-carbon lyase activity	21
Molecular function	Carboxy-lyase activity	14
Biological process	Glutamine family amino acid metabolic process	12
Biological process	Energy coupled proton transport, down electrochemical gradient	9
Biological process	ATP synthesis coupled proton transport	9
Molecular function	Carboxylic acid binding	7
Molecular function	Organic acid binding	7
Molecular function	Transferase activity, transferring acyl groups, acyl groups converted into alkyl on transfer	6
Mmolecular function	Amino acid binding	6
Biological process	Pyridine-containing compound biosynthetic process	6
Molecular function	Proton-transporting ATP synthase activity, rotational mechanism	5
Molecular function	ATPase activity, coupled to transmembrane movement of ions, rotational mechanism	5
Molecular function	Cation-transporting ATPase activity	5
Molecular function	ATPase activity, coupled to transmembrane movement of ions	5
Biological process	Organic hydroxy compound biosynthetic process	5

**Depleted**		

**Term Type**	**Description**	**DEG**

Biological process	Bbiological regulation	196
Molecular function	Cation binding	191
Biological process	Regulation of cellular process	190
Biological process	Regulation of biological process	190
Molecular function	Metal ion binding	187
Molecular function	Transition metal ion binding	161
Molecular function	Zinc ion binding	122
Molecular function	Nucleic acid binding transcription factor activity	87
Molecular function	Transcription factor activity, sequence-specific DNA binding	87
Molecular function	RNA polymerase II transcription factor activity, sequence-specific DNA binding	60
Molecular function	Heme binding	39
Mmolecular function	Tetrapyrrole binding	39
Molecular function	Iron ion binding	36
Molecular function	Oxidoreductase activity, acting on single donors with incorporation of molecular oxygen	13
Biological process	Siroheme biosynthetic process	12
Molecular function	Precorrin-2 dehydrogenase activity	12
Biological process	Siroheme metabolic process	12
Molecular function	Ooxidoreductase activity, acting on peroxide as acceptor	10
Molecular function	Peroxidase activity	8

*DEG is the number of differentially expressed genes induced by Al in the absence of the caryopsis. The darker the DEG cell, the higher the number of DEGs.*

**TABLE 11:** KEGG pathway terms associated with Al exposure in the absence of the caryopses (FOA).

Term	DEG
Biosynthesis of secondary metabolites	108
Biosynthesis of amino acids	64
Carbon metabolism	44
2-Oxocarboxylic acid metabolism	24
Cysteine and methionine metabolism	20
Valine, leucine and isoleucine degradation	18
Propanoate metabolism	13
Lysine biosynthesis	11

*DEG is the number of differentially expressed genes induced by Al in the absence of the caryopsis. The darker the DEG cell, the higher the number of DEGs.*

### Gene Ontology Enrichment Is Influenced by Caryopsis Colonization During Aluminum Exposure

Caryopsis colonization during aluminum exposure (FA) resulted in the induction of gene ontology terms associated with biological, cellular component, and molecular functions. These gene ontology terms were associated with some general biological processes, including ion binding, transport, membrane-related functions, oxidoreductase activity, and lipid metabolic processes (Table 12).

**TABLE 12:** Enriched gene ontology terms associated with Al exposure in the presence of caryopses (FA vs. FOA).

Term Type	Description	DEG
Biological process	Single-organism process	174
Biological process	Single-organism cellular process	110
Cellular component	Membrane	107
Molecular function	Ion binding	82
Biological process	Localization	71
Biological process	Transport	69
Biological process	Establishment of localization	69
Cellular component	Membrane part	69
Cellular component	Integral component of membrane	63
Cellular component	Intrinsic component of membrane	63
Biological process	Single-organism localization	59
Biological process	Single-organism transport	58
Molecular function	Anion binding	48
Biological process	Transmembrane transport	40
Molecular function	Transporter activity	40
Molecular function	Transmembrane transporter activity	37
Biological process	Lipid metabolic process	32
Molecular function	Oxidoreductase activity, acting on paired donors, with incorporation or reduction of molecular oxygen	15
Molecular function	Flavin adenine dinucleotide binding	15
Molecular function	Monooxygenase activity	7
Molecular function	Protein tyrosine phosphatase activity	5
Molecular function	Phosphoprotein phosphatase activity	5
Biological process	Protein dephosphorylation	5
Biological process	Amino acid transmembrane transport	4
Biological process	Anion transmembrane transport	4
Biological process	Organic acid transmembrane transport	4
Molecular function	Protein tyrosine/serine/threonine phosphatase activity	4
Molecular function	N,N-dimethylaniline monooxygenase activity	4
Cellular component	Photosystem II reaction center	4
Molecular function	O-acyltransferase activity	4
Molecular function	Primary amine oxidase activity	2
Molecular function	Calcium-dependent phospholipid binding	2
Molecular function	Sodium:proton antiporter activity	1
Molecular function	Glycogen (starch) synthase activity	1
Biological process	Glycogen metabolic process	1
Biological process	Glycogen biosynthetic process	1
Biological process	Energy reserve metabolic process	1
Biological process	Cobalamin transport	1
Biological process	Vitamin transport	1
Molecular function	Calcium activated cation channel activity	1
Molecular function	Calcium-activated potassium channel activity	1
Molecular function	Ion gated channel activity	1
Cellular component	Acetyl-CoA carboxylase complex	1
Molecular function	Acetyl-CoA carboxylase activity	1
Molecular function	CoA carboxylase activity	1
Molecular function	Ligase activity, forming carbon-carbon bonds	1

*DEG is the number of differentially expressed genes induced by Al in the absence of the caryopsis. The darker the DEG cell, the higher the number of DEGs.*

### KEGG Pathway Analysis During Caryopsis Colonization

Caryopsis colonization (in the absence of Al) was associated with an increase in 23 DEGs associated with carbon metabolism, and an increase in 10 DEGs associated with the tricarboxylic acid cycle (TCA cycle). Additionally, a decrease in 5 DEGs associated with N-Glycan biosynthesis was observed in fungal tissues samples colonizing *A. fatua* caryopses.

## Discussion

Caryopsis colonization resulted in induction of F.a.1 genes associated with virulence, stress/defense, detoxification, organic acid metabolism, basic metabolism, transport, and amino acid/peptide/protein metabolism. At the same time, repression of genes associated with iron metabolism, stress/defense, organic acid metabolism, metal-related metabolism, and basic metabolism was observed. These results suggest a shift in gene expression related to fundamental biological functions occurs during caryopsis colonization. It should be noted that future researchers might consider sampling fungal tissues in a spatial manner that includes sampling the zone of fungal material directly in contact with the caryopsis separately from the remaining hyphal mat. A more spatially refined sampling method might also reveal more information with respect to GO enrichment and KEGG pathway analyses at specific interaction sites.

Nevertheless, the presented results reveal many biological mechanisms associated with caryopsis colonization. Notably, AKR7L: Aflatoxin B1 aldehyde reductase member 4 was strongly induced, suggesting a role for aflatoxin degradation during *F. avenaceum* caryopsis colonization. Previous work suggests *Fusarium* and *Aspergillus* species (which produce aflatoxins) can be the primary fungi associated with post-harvest mycotoxin contaminated wheat and corn (Ali et al., 1998; Del Palacio et al., 2016). Several oxidoreductase genes were induced during caryopsis colonization (Table 1), which is of relevance because the *F. avenaceum* genome has been shown to be enriched in oxidoreductase genes, several of which were induced during colonization of barley plants (Lysøe et al., 2014). Additionally, in the current study, many genes related to oxidative stress were induced during caryopsis colonization, including peroxisomal catalase and catalase-1 (cat-1). Others have found *Fusarium* isolates with a greater capacity to cope with oxidative stress also exhibit stronger virulence (Ponts et al., 2009). Together these results suggest oxidoreductase genes are key to *F. avenaceum* pathogenicity of both seeds and developed plant tissues, likely through mitigating oxidative stress. Further overlap was observed between the results of the current study and those of Lysøe et al., 2014, including induction of the same or related genes, such as, NADH-related genes, an ATP synthase subunit, an extracellular serine-rich protein, heat shock proteins, an iron-sulfur cluster assembly protein, cytochrome p450, and others (Table 1 and Supplementary Table S1). Heat shock protein 90 is required for virulence and development in *F. graminearum* (Bui et al., 2016), and while this heat shock protein was not induced during caryopsis colonization in the current study, several others were, with the highest expression observed in HSP31 (Table 1); this suggests a role for other heat shock proteins in the virulence of *Fusarium* species. Other virulence-related genes induced in the current study included ga4 desaturase and ent-kaurene oxidase; both are involved in the production of gibberellic acid, which is also thought to play a role in fungal pathogenesis (Malonek et al., 2005; Chanclud and Morel, 2016).

Many differentially induced genes were unique to the present study as compared with Lysøe et al. (2014), including those involved in the metabolism of the organic acids, oxalate and malate. Additionally, nitrogen metabolism genes were induced during caryopsis colonization, including urea amidolyase and nitrite reductase. Many genes related to carbon metabolism were induced during caryopsis colonization, including *STL1* (a sugar transporter), *ght1* (a glucose transporter), *glcA* (glucan endo-1,3-beta-glucosidase A1), *grg-1* (glucose-repressible gene protein), and *Gpd2* (glycerol-3-phosphate dehydrogenase). In studies with *F. graminearum*, *STL1* has been shown to be involved in interactions with living verses dead wheat tissues, and is thought to be induced by plant signals (Boedi et al., 2016). While the precise role of *grg-1* remains unknown in *Fusarium* species, it is known that glucose repression is intimately linked with fungal-driven cell wall degradation and is necessary for virulence (Tonukari et al., 2000; Ospina-Giraldo et al., 2003). The results suggest a role of *grg-1* in *A. fatua* caryopsis colonization. The *gpd2* gene has been shown to be necessary for glycerol utilization, and deletion of the gene results in reduced virulence of *Pyricularia oryzae* (Shi et al., 2018). Several phosphatase genes were induced during caryopsis colonization, which is of relevance because not only is phosphate turnover central to basic biological functions, but phosphatases have also been shown to be crucial to virulence in *F. graminearum* (Yun et al., 2015). Another stress-related gene that was induced during caryopsis colonization was *sed1*, which is thought to be involved in cell wall stability (Hagen et al., 2004).

Bioavailable Al is thought to play a major role in inhibition of many plant pathogenic fungi, including inhibition of virulence and macroconidial germination of *Fusarium solani* f. sp. *phaseoli* (Firestone et al., 1983; Kobayashi and Ko, 1985; Meyer et al., 1994; Furuya et al., 1999; Fichtner et al., 2006). Chronic exposure of F.a.1 to sublethal concentrations of Al in the current study resulted in global transcriptomic changes (Figures 3E,F). Notably, Al exposure led to induction of siderophore-, iron-, and organic acid-related genes. Fungal-derived siderophores and organic acids are known to interact with metals (Renshaw et al., 2002; Sullivan et al., 2012). Aluminum exposure-induced alterations in iron metabolism included induction and repression of genes associated with iron transport, suggesting a general disruption in iron metabolism in response to Al exposure. Several genes involved in siderophore biosynthesis were induced during Al exposure, including *sidA* (L-ornithine N(5)-monooxygenase), which is crucial for viability of *Aspergillus nidulans* (Eisendle et al., 2003), and is required for full virulence of *F. graminearum* (Greenshields et al., 2007). The siderophore biosynthesis genes, *sidD* (*NRPS4*) and *sidC* (*NRPS2*) were also induced in response to Al, and are known to be responsible for synthesis of fusarinine C and ferricrocin, respectively, in *Aspergillus fumigatus* (Schrettl et al., 2007). In both *Cochliobolus heterostrophus* and *F. graminearum* (*Gibberella zeae*), *sidC* has been found essential for sexual development, with the phenotype being partially restored in knockout mutants supplemented with iron (Oide et al., 2007). Reduced colony forming unit counts were observed in *ΔsidA* and *ΔsidD* mutants of *A. fumigatus* used to test the role of fungal siderophores in infecting mice corneas (Leal et al., 2013). Additionally, the transporter, *mirB* was induced in response to Al exposure. It has been shown that *mirB* is involved exclusively in transporting triacetylfusarinine C (Haas et al., 2003), which has been implicated as playing key roles in iron uptake and virulence of *F. graminearum* (Oide et al., 2015). Another siderophore transporter, *mirA*, was also induced by Al toxicity, which has been shown to exclusively transport enterobactin, a bacterial siderophore (Haas et al., 2003). The siderophore transporter *sit1*, was also induced by Al exposure. Greenshields et al. (2007), found *sit1* was induced by low iron, but was not expressed in infected wheat, suggesting a role in iron metabolism, but not necessarily for virulence. Another siderophore transporter that was induced during Al toxicity was *str3*, which is known to be negatively regulated by iron (Pelletier et al., 2003), and is thought to be crucial for low-affinity heme acquisition by *Schizosaccharomyces pombe* (Normant et al., 2018). A transcript having 93.4% sequence similarity to *besA*, ferri-bacillibactin esterase, was also induced in response to Al exposure. The *besA* sequence has also been identified in *F. oxysporum* (Guo et al., 2014), while, in *Bacillus* species, the besA protein is involved in liberating iron from Fe-bacillibactin complexes (Abergel et al., 2009). In the context of the findings presented here, an analogous function for the protein may occur in fungal species, as well.

Organic acids are known to play important roles in metal availability through complexation with metal ions, which renders the metal less bioavailable (Jones, 1998), and the phenomenon has been observed in fungi. For instance, oxalic acid production is responsible for zinc and copper tolerance in *Aspergillus niger* and *Penicillium citrinum*, and *A. niger* isolated from lead contaminated soils has been found to secrete large amounts of organic acids (Sullivan et al., 2012; Sazanova et al., 2015). Additionally, malate exudation by *Penicillum oxalicum* was suggested to be involved in phosphate liberation from AlPO_4_, FePO_4_, and Ca_3_(PO_4_)_2_ (Gadagi et al., 2007). These findings, along with the fact that malate is thought to be responsible for Al tolerance in plants (Delhaize and Ryan, 1995; Ryan et al., 1995; Klugh-Stewart and Cumming, 2009), suggests the induction of malate transporters observed in F.a.1 exposed to Al is likely related to modulating Al bioavailability.

Gene ontology and KEGG pathway analyses also showed Al exposure was associated with alterations in basic biological processes, including induction of genes related to oxidoreductase activity, oxidation-reduction process, organic acid metabolism, biosynthesis of secondary metabolites, biosynthesis of amino acids, carbon metabolism, 2-oxocarboxylic acid metabolism, and cysteine and methionine metabolism. Alterations in these disparate biological processes suggests Al toxicity results in changes to the basic metabolism of the fungus. Gene ontology analyses also suggest that uniquely expressed DEGs might also be involved in unique biological processes (Supplementary Table S7), however, these data were filtered to remove GO terms with less than 5 DEGs to identify potentially unique biological functions associated with the uniquely expressed genes. The unfiltered results showing the number of unique DEGs associated with GO terms can be found in Supplementary Table S8.

These results have significance regarding the currently expanding global issue of soil acidification, and associated Al toxicity. It is unclear how soil fungi, particularly pathogens, cope with acid soils and the metals that become toxic at lower pH levels in acidified soils, at the molecular level. The results presented here suggest siderophores and organic acids are likely involved in Al toxicity responses in F.a.1. Which, in turn, brings attention to the fact that these interactions in soils and their impacts on weed seed decay have been overlooked to date. The transcriptomic responses of F.a.1 to Al suggest that Al toxicity results in dramatic changes in iron metabolism, siderophore metabolism, and organic acid metabolism, simultaneously. This is a key finding because iron homeostasis has been shown to be essential for full virulence of the related pathogenic fungus, *F. oxysporum* (López-Berges et al., 2012). Additionally, siderophore production is known to be important in plant-pathogen interactions, including the pathogenic activity and sexual development of *F. graminearum* (Greenshields et al., 2007; Oide et al., 2015). A future direction might include examining the influence of Al (and other metals) on competitiveness and virulence of F.a1 in soils.

The transcriptome of F.a.1 provided insights regarding the interactions between an oat fungal pathogen, its susceptible host, and aluminum challenge. A complex hierarchy of gene expression produced by aluminum challenge, superseded that of the host interaction. Understanding genes involved in Al tolerance may be utilized to promote fungal activity under high Al soil conditions. Additionally, better understanding of fungus-Al interactions could also assist identification and development of plant growth-promoting fungi that can tolerate increased Al bioavailability. Key genes associated with pathogen growth and life cycle, including the formation of spores, conidia, and infection structures were revealed (Table 6 and Supplementary Tables S2, S3). These genes are promising candidates for host-induced gene silencing (HIGS), an RNAi-based approach for pathogen suppression that has been deployed against *Fusarium* spp. (reviewed in Okubara et al., 2019). Additionally, these genes (and others identified here) may be utilized in identifying other fungal strains that may be even more effective in causing weed seedbank decay. Future work might include applying a multi-omic approach to further elucidate and clarify molecular mechanisms of caryopsis colonization and aluminum toxicity in *F. avenaceum*, including metabolomic and proteomic approaches. Additionally, future work could aim to examine the subcellular localization of the proteins involved in caryopsis colonization and Al toxicity to reveal how Al and seed colonization influence the subcellular organization of important metabolites, proteins, and metals.

## Data Availability Statement

The RNA sequence datasets generated for this study can be found through the Sequence Read Archive (SRA; SRA accession: PRJNA595343).

## Author Contributions

RL conceived and performed the experiments, analyzed the data, and was the primary author of the manuscript. PO assisted with experimental design, data analysis, and writing of the manuscript. EF assisted with experimental design, determined PPO activity, and assisted with editing the manuscript. RH assisted with experimental design, extracted RNA, and provided text regarding the RNA extraction protocol. DG assisted with experimental design. TS assisted with experimental design and editing the manuscript.

## Conflict of Interest

The authors declare that the research was conducted in the absence of any commercial or financial relationships that could be construed as a potential conflict of interest.
